# Roles of peptidylarginine deiminase (PAD) and protein citrullination in viral infections

**DOI:** 10.3389/fmicb.2026.1792839

**Published:** 2026-03-31

**Authors:** Kaili Wang, Ting Ma, Minghui Ouyang, Xiao Fei, Ling'en Yang, Guijie Guo

**Affiliations:** 1 Key Laboratory of Animal Pathogen Infection and Immunology of Fujian Province, College of Animal Sciences, Fujian Agriculture and Forestry University, Fuzhou, China; 2 Joint Laboratory of Animal Pathogen Prevention and Control of Fujian-Nepal, College of Animal Sciences, Fujian Agriculture and Forestry University, Fuzhou, China; 3 Engineering Research Center for Animal Breeding and Sustainable Production, College of Animal Sciences, Fujian Agriculture and Forestry University, Fuzhou, China; 4 Key Laboratory of Fujian-Taiwan Animal Pathogen Biology, College of Animal Sciences, Fujian Agriculture and Forestry University, Fuzhou, China

**Keywords:** citrullination, PAD, PAD activator, PAD inhibitor, viral infection

## Abstract

Peptidylarginine deiminases (PADs) are calcium-dependent enzymes that catalyze protein citrullination, a post-translational modification implicated in both physiological processes and the pathogenesis of various diseases. This review provides a systematic overview of the PAD family's roles in viral infections, with a focus on their tissue distribution, activation mechanisms, and functional significance in host-pathogen interactions. Accumulating evidence indicates that diverse viruses have evolved to exploit specific host PAD isoforms as a strategy to subvert antiviral immunity and enhance viral replication. This review delves into the intricate interplay between viruses and the PAD family, detailing how distinct viruses selectively upregulate or hijack particular PAD isoforms to disrupt host immune defenses. We further compare the divergent strategies employed by DNA and RNA viruses in leveraging PAD-mediated citrullination and summarize the therapeutic potential of PAD inhibitors, such as GSK484 and Cl-amidine, in antiviral interventions. Additionally, demethoxycurcumin (DMC), as the only identified selective small-molecule activator of PAD2 to date, provides a valuable tool for investigating the role of PAD2-mediated citrullination in viral infection. Finally, we discuss current research limitations and propose future directions aimed at deciphering the complex virus-PAD-host axis, with the ultimate goal of informing the development of novel antiviral therapeutics.

## 1 Introduction

**Figure 1:**
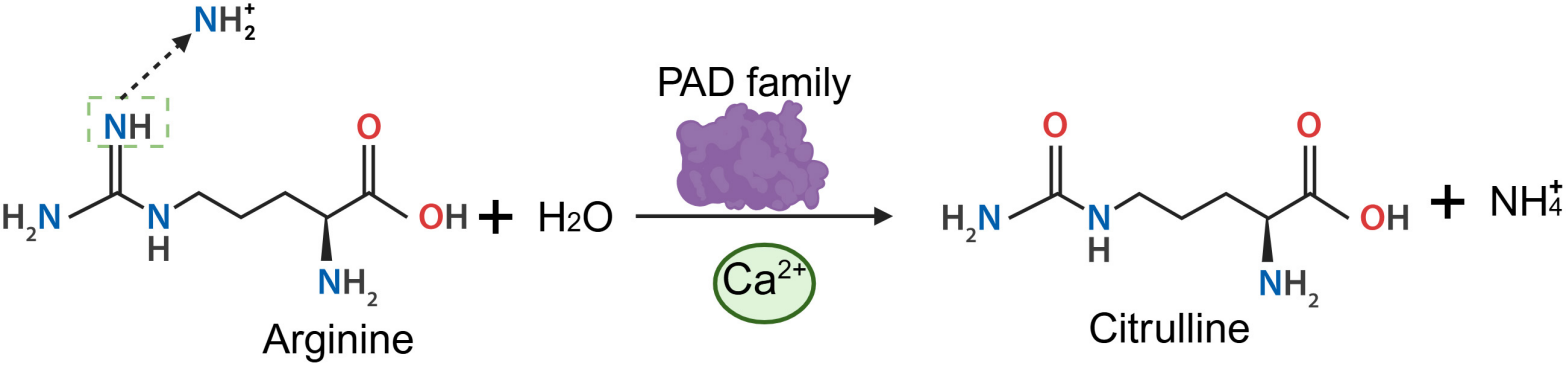
A schematic diagram of protein citrullination. PAD enzymes convert arginine into citrulline bearing a ureido group (–NH–CO–NH–) by hydrolyzing the guanidino group of arginine and removing an imino group (–NH). Created with BioRender (BioRender.com).

Protein citrullination is a post-translational modification (PTM) of proteins catalyzed by peptidylarginine deiminase (PAD), which converts peptidylarginine into peptidylcitrulline in a process also known as deimination (Figure 1) (Zhu et al., 2021; Ciesielski et al., 2022; Yu and Proost, 2022; Barasa and Thompson, 2023). This modification is irreversible and calcium-dependent. Under physiological conditions, a significant gradient exists between extracellular (1.0–1.3 mM) and intracellular (10^−5^ to 10^−3^ mM) calcium concentrations (Quist et al., 2000; György et al., 2006). Calcium ion concentrations of 10^−2^ mM have been shown to activate PADs, which is far exceed the intracellular calcium ion levels observed under normal physiological conditions. Consequently, substantial calcium ion influx activates protein citrullination and promotes PADs activation. This calcium can originate from either the extracellular space or intracellular stores (Hensen and Pruijn, 2014; Alghamdi et al., 2019). Disruption of calcium homeostasis, such as during cell death or certain pathological states, elevated calcium ion concentrations activate PAD, leading to the citrullination of extracellular proteins (Schwab et al., 2002).

**Figure 2:**
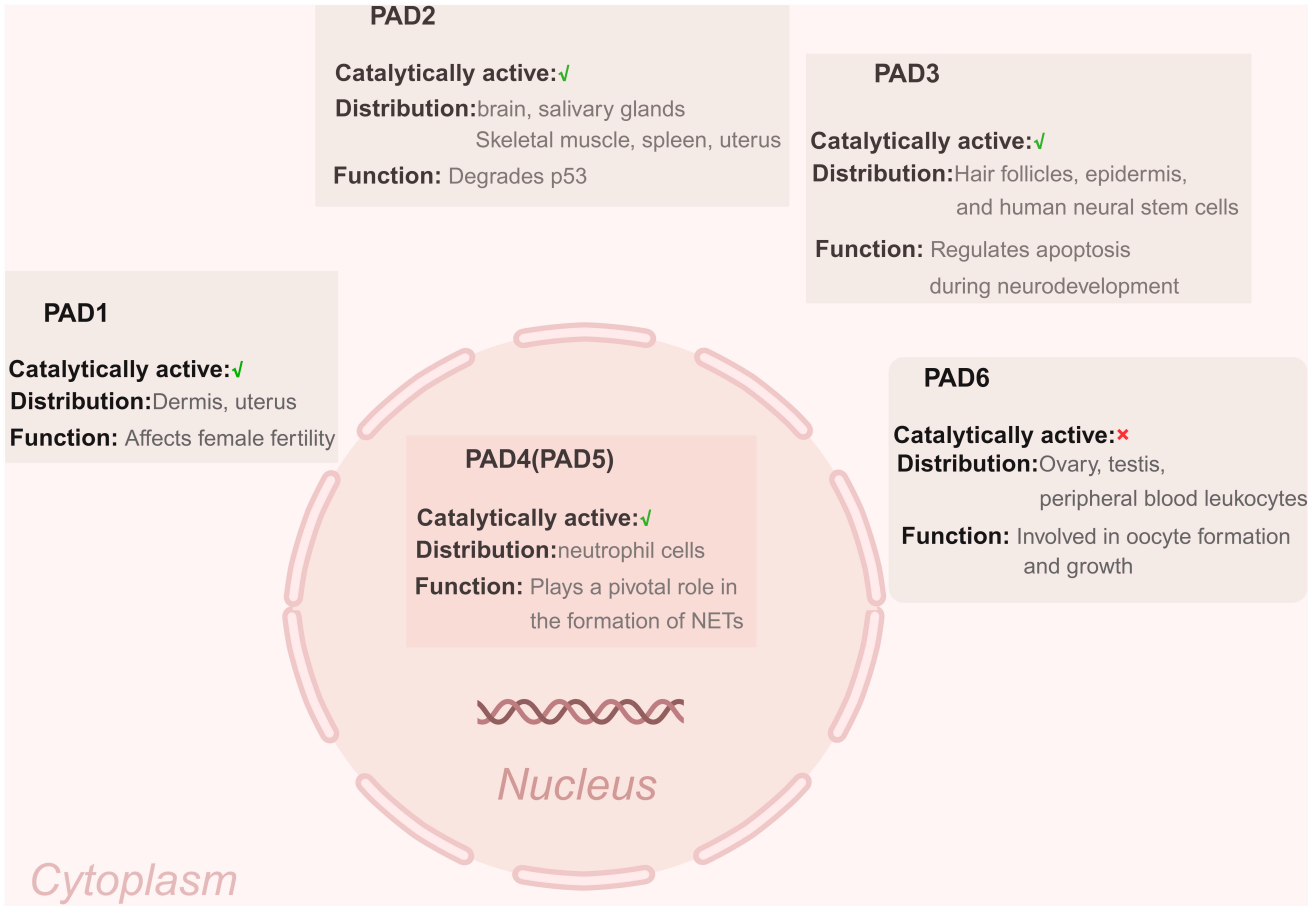
The PADs with distinct catalytic activity, localization and functions. The PAD enzymes PAD1, PAD2, PAD3, and PAD6 are found in the cytoplasm (with PAD6 being catalytically inactive), whereas PAD4/PAD5 is localized within the nucleus. Created with BioRender (BioRender.com).

The PAD family comprises five isoforms: PAD1–4 and PAD6 (Bicker and Thompson, 2013; Mondal and Thompson, 2019, 2021). However, only PAD1–4 are catalytically active. PAD6 is catalytically incompetent due to a mutation in its active site (Yu and Proost, 2022). PAD1–3, and PAD6 are cytosolic, while PAD4 is localized in the nucleus (Nava-Quiroz et al., 2023). Their tissue distribution and functions are also specialized. PAD1 is highly expressed in the epidermis and uterus, where it influences female fertility (Yu and Proost, 2022). The expression of the PAD2 gene is primarily observed in the brain, salivary glands, skeletal muscle, spleen, and uterus, with specific physiological functions attributed to its expression in distinct tissues (Nava-Quiroz et al., 2023; Zhang et al., 2025). Research indicates that the tumor suppressor protein p53 can be degraded by PAD2, and the degradation of p53 has been shown to reduce the likelihood of cell cycle arrest and apoptosis (Yu and Proost, 2022). Additionally, both PAD2 and PAD4 are implicated in various solid tumors (Guo and Fast, 2011; Wang and Wang, 2013; Yu and Proost, 2022). PAD3 is primarily distributed in hair follicles and epidermal tissues, as well as in human neural stem cells where it modulates apoptosis during neural development through calcium-dependent mechanisms (Wang and Wang, 2013; Yu and Proost, 2022). PAD4, previously referred to as PAD5, is widely expressed, especially in neutrophils (Yu and Proost, 2022). PAD6 is distributed in the ovaries, testes and peripheral blood leukocytes. Furthermore, the role of PAD6 in oocyte maturation and growth is highly significant (Yu and Proost, 2022; Nava-Quiroz et al., 2023) (Figure 2).

Citrullination is associated with numerous diseases, including rheumatoid arthritis (RA), multiple sclerosis (MS), lupus erythematosus (LE), Alzheimer's disease (AD), Parkinson's disease (PD), interstitial lung disease (ILD), ulcerative colitis (UC) and cancer. Additionally, it also plays a crucial role in viral infections (Casanova et al., 2020; Radermecker et al., 2020; Griffante et al., 2021; Pasquero et al., 2023, 2024; Ishigami et al., 2005; Kearney et al., 2005; Moscarello et al., 2007; Chang et al., 2009; Mondal and Thompson, 2019; Nava-Quiroz et al., 2023). Viruses have evolved to subvert host antiviral defenses through several key mechanisms. A major strategy is the manipulation of PAD-mediated protein citrullination. Different viral infections induce distinct alterations in PAD expression. For example, SARS-CoV-2 infection induces cell-specific changes in PAD expression (Radermecker et al., 2020). Here, PAD4 is involved in promoting neutrophil extracellular trap (NET) accumulation and thrombosis, as well as regulating coronavirus replication (Adriana Gutiérrez-Pérez et al., 2025). Conversely, PAD2 exhibits more pronounced pro-fibrinogen citrullination activity (van Beers et al., 2010; Damgaard et al., 2018). During human cytomegalovirus (HCMV) infection, PAD-mediated citrullination of interferon-stimulated genes (e.g., IFIT1) weakens the antiviral function of their products. This modification disrupts IFIT1's binding to viral RNA and clears obstacles for HCMV replication and transmission (Griffante et al., 2021). Human rhinovirus (HRV) evades the host defense by citrullinating LL-37, a critical antimicrobial peptide, thereby neutralizing its antiviral and anti-inflammatory functions. This modification directly weakens the innate immune barrier function of the respiratory mucosa, creating a favorable environment for HRV to spread further within the host (Casanova et al., 2020). Herpes simplex virus type 1 (HSV-1) selectively activates PAD3, triggering the concurrent citrullination of two key effector proteins, IFIT1 and IFIT2, in the interferon pathway. This modification potently suppresses their function, thereby blocking the host's interferon-mediated antiviral response and ultimately enabling HSV-1 to evade immune surveillance (Pasquero et al., 2023). Similar to HSV-1, HSV-2 infection upregulates host PAD3 expression and activity, thereby adding intracellular protein citrullination to support viral replication. This represents a conserved strategy across these related viruses.

While previous reviews have described the roles of PADs and citrullination in inflammation and autoimmune diseases, few have systematically integrated these processes with viral infection. This review extends beyond summarizing known associations among PADs, citrullination, and viral infections by proposing a unifying conceptual framework, which centers on citrullination as a common regulatory node linking viral replication, host innate immune responses, immune evasion, and inflammatory pathology. Rather than merely listing virus-specific observations, we synthesize viral infection mechanisms, highlight key unanswered questions, and clarify the role of PAD-mediated citrullination in both antiviral defense and virus-driven immunopathology, thereby providing a novel synthetic perspective distinct from existing virological and immunological reviews.

## 2 Molecular mechanisms governing PADs activation: regulation, and catalysis

**Figure 3:**
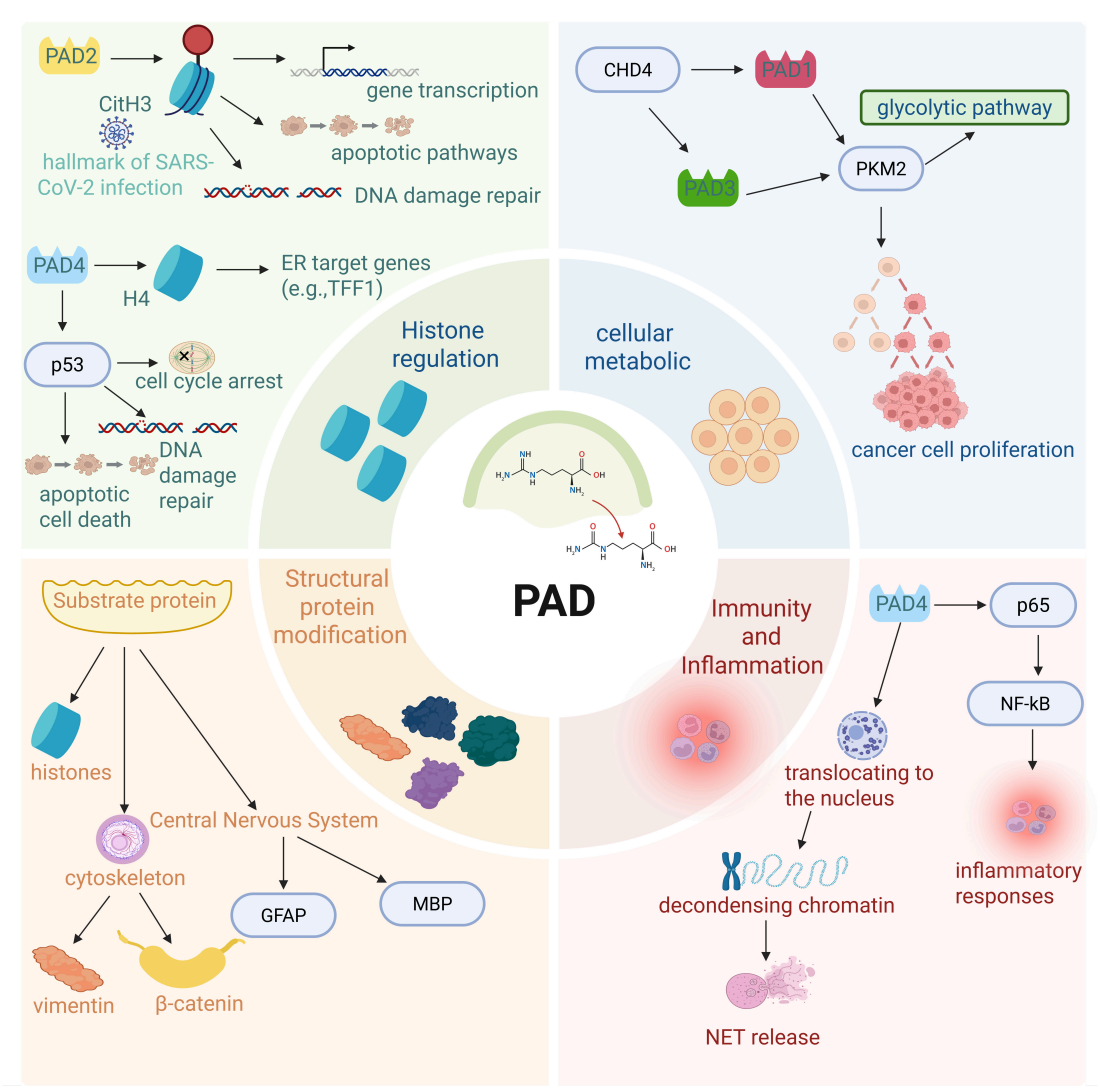
Cellular regulatory networks mediated by substrate-specific citrullination of PAD isoforms. This schematic summarizes core cellular functions and disease-associated pathways regulated by multiple PAD isoforms through the citrullination of specific substrates. (i) Histone modifications: PAD2 catalyzes the citrullination of histone H3 to form CitH3, a hallmark of SARS-CoV-2 infection linked to the regulation of gene transcription, DNA damage repair, and apoptotic pathways. PAD4 primarily mediates histone H4 citrullination, modulating the expression of estrogen receptor target genes (e.g., TFF1) and the p53 pathway, thereby influencing cell cycle arrest, DNA repair, and apoptosis. (ii) Structural protein modifications: The citrullination of cytoskeletal proteins (e.g., vimentin and β-catenin) and central nervous system-specific proteins (e.g., GFAP and MBP) by PAD enzymes is crucial for regulating cell morphology and maintaining neural structure and function, respectively. (iii) Metabolic and proliferative regulation: The chromodomain helicase DNA-binding protein 4(CHD4) controls the levels of PAD1 and PAD3, which in turn regulate glycolysis and cancer cell proliferation through citrullination of PKM2. (iv) Inflammatory and immune responses: PAD4 modulates inflammatory responses by activating NF-κB and facilitating its nuclear translocation, and triggers NETs release. Created with BioRender (BioRender.com).

PADs play substantial roles in diverse cellular processes, including encompassing myelination, gene transcription, kinase signal transduction, cutaneous keratinization, antigen generation, apoptosis, regulation of reproductive function, the formation of NETs and macrophage extracellular traps (Figure 3). Among the PAD family, PAD2 facilitates the citrullination of multiple proteins, for instance β-catenin, histones, glial fibrillary acidic protein (GFAP), myelin basic protein (MBP), and vimentin (Jones et al., 2009; Falcão et al., 2019; Wang et al., 2021; Yu and Proost, 2022). Notably, histones are thought to be the primary and most characteristic substrates of PAD2 (Ishigami et al., 2005; Kearney et al., 2005; Moscarello et al., 2007). In animal models, following SARS-CoV-2 infection, elevated levels of citrullinated histone 3 imply that such citrullinated proteins could function as indicators of SARS-CoV-2 infection (Adriana Gutiérrez-Pérez et al., 2025). Furthermore, evidence has demonstrated that PAD2 modulates various cellular processes through CitH3, including gene transcription, DNA damage repair, and apoptotic pathways (Zhang et al., 2012; Dwivedi et al., 2014; Yu and Proost, 2022). Furthermore, citrullination also regulates protease activity, with a multitude of matrix metalloproteinases (MMPs) identified as PAD substrates (Boon et al., 2021; Grillet et al., 2021). The processes of transcriptional regulation and the DNA damage response are linked to histone citrullination (Christophorou et al., 2014). The regulation of chromatin accessibility by citrullination at different sites exhibits specificity. For instance, PAD4-catalyzed citrullination at H3R2 and H3R8 directly disrupts the binding of histone H3 to DNA, promoting nucleosome dissociation (Wang et al., 2009). H3R17 citrullination further weakens the interaction between histones and chromatin remodeling complexes, accelerating chromatin depolymerization and creating spatial conditions for transcription factor binding (Love et al., 2025). On the contrary, in specific cellular contexts, histone citrullination can also promote chromatin condensation through indirect mechanisms: when citrullination occurs at binding sites between histones and repressive chromatin factors (such as heterochromatin protein 1α, HP1α), it enhances HP1α binding to chromatin, induces heterochromatin formation, and reduces chromatin accessibility (Love et al., 2025). This mechanism plays a crucial role in viral latency regulation. *In vitro* studies have shown that PAD4 is involved in regulating the p53 pathway, a key pathway required for cell cycle arrest, apoptotic cell death, and DNA damage repair (Guo and Fast, 2011). By translocating to the nucleus, decondensing chromatin, and driving NET release, PAD4 exerts a pivotal function in NET formation. The NETs can sequester and eliminate microorganisms, thereby supporting pathogen clearance processes.

Coassolo et al. (2021) identified chromodomain helicase DNA-binding protein 4 (CHD4) as a critical regulator of cellular metabolic processes. *In vitro* studies indicate that CHD4 controls the abundance of PAD1 and PAD3, which in turn affects the citrullination of pyruvate kinase M2 (PKM2) (Coassolo et al., 2021). This modification is significant for the regulation of the glycolytic pathway and cancer cell proliferation.

In the context of viral infections, PAD participates in a spectrum of inflammatory and autoimmune responses while regulating the release of extracellular vesicles (EVs). The extent of its involvement in infection and immune modulation has been partially elucidated. Studies demonstrate that HRV infection induces the upregulation of PAD activity, and citrullination of the human cathelicidin LL-37 abrogates LL-37's ability to counteract HRV infection; this observation confirms that citrullination serves as a viral evasion mechanism utilized by specific viruses (Casanova et al., 2020). PAD activity exerts control over inflammatory responses by adjusting cytokine levels. For instance, during viral infections, PAD4 modulates nuclear factor-κB (NF-κB) signaling via p65 citrullination, which in turn elicits inflammatory responses (Yu and Proost, 2022). Furthermore, PAD4 and elevated histone citrullination participate in the formation of NETs; their overexpression enhances viral infections (Wang and Wang, 2013).

## 3 PADs and protein citrullination in viral infections: molecular mechanisms, and pathological implications

Viruses of diverse families, including Coronaviridae, Herpesviridae, Picornaviridae, and Papillomaviridae, have evolved distinct strategies to exploit PADs for their own replication and pathogenicity, with shared and unique mechanisms centered on immune escape, chromatin remodeling, NET formation, and regulation of viral replication stages. A key shared mechanism underlying these interactions is the selective upregulation of specific PAD isoforms during infection: SARS-CoV-2 specifically upregulates PAD4 transcription and expression in host cells without significant effects on other PADs, thereby creating a prerequisite for its preferential utilization of PAD4 (Pasquero et al., 2022); HCMV upregulates PAD2 and PAD4 during early infection via transcriptional activation by the immediate early protein IE1 (Griffante et al., 2021; Pasquero et al., 2023); HRVs induce significant upregulation of PAD enzymes in bronchial epithelial cells and peripheral blood mononuclear cells, with PAD2 showing the most prominent increase (Mello et al., 2014; Li et al., 2024); HSV-1 and HSV-2 upregulate PAD2, PAD3, and PAD4 following infection, with PAD3 exhibiting the most significant upregulation and synchronizing with the HSV-1 early viral protein ICP27 (Pasquero et al., 2024); and HPV-infected patients show significantly elevated PADI4 expression, which progressively increases with disease severity in HPV-associated cervical lesions (Chang et al., 2009; Albano et al., 2024). This selective upregulation of PAD isoforms lays the foundation for subsequent viral manipulation of host cellular processes through PAD-mediated citrullination.

### 3.1 Immune escape

Among the core mechanisms by which viruses exploit PADs, immune escape stands out as a central strategy, whereby multiple viruses utilize PADs and PAD-mediated citrullination to evade host antiviral responses. For instance, HCMV induces the citrullination of IFIT1, a process that impairs its antiviral function and enhances viral proliferation-an effect demonstrated by increased HCMV replication in IFIT1 knockout mouse models (Griffante et al., 2021; Pasquero et al., 2023). Similarly, HSV-1 exploits PAD3-catalyzed citrullination to disrupt the spatial structure and function of IFIT1/IFIT2, thereby impairing their ability to inhibit viral replication (Pasquero et al., 2024). SARS-CoV-2 also leverages PADs for immune evasion, preferentially utilizing PAD4 due to the high compatibility between PAD4 substrates and the virus's requirements for evading host immunity (Pasquero et al., 2022), while HRV hijacks PADs to interfere with the antiviral and anti-inflammatory functions of the host defense peptide LL-37, further facilitating immune escape (Casanova et al., 2020). HPV, particularly high-risk subtypes, relies on PAD-mediated disruption of the p53-p21 regulatory pathway: pan-PAD inhibitors such as BB-Cl-amidine downregulate HPV E6/E7 expression, restore p53 and p21 levels, and thereby reduce HPV-driven carcinogenic potential (Scarth et al., 2021; Albano et al., 2024). Additionally, HCMV-infected plasmacytoid dendritic cells (PDCs) activate B cell proliferation and differentiation, a process linked to PADs-mediated regulation of B cell function and the generation of virus-specific antibodies (Liu et al., 2018; Griffante et al., 2021), which further contributes to viral immune evasion.

### 3.2 Chromatin remodeling and NET formation

Another critical mechanism contributing to viral pathogenesis involves chromatin remodeling and NET formation, processes primarily mediated by PAD4 across multiple viral infections. In the case of SARS-CoV-2, PAD4 upregulation induced by the virus catalyzes the citrullination of specific arginine residues on histone tails; this modification neutralizes arginine's positive charge, leading to chromatin relaxation and subsequent NET deposition. This process is not only critical to SARS-CoV-2-induced severe acute respiratory syndrome but also represents a key target for therapeutic intervention (Radermecker et al., 2020; Mondal and Thompson, 2021). PAD4, which exhibits dual nuclear and cytoplasmic localization, participates in chromatin regulation within the nucleus, and this nuclear localization overlaps with that of HPV early proteins (E6/E7) to facilitate their interaction (Chang et al., 2009; Albano et al., 2024). HCMV also leverages PAD4-mediated NET formation: PAD4-driven citrullination induces NET formation and the production of pro-inflammatory cytokines, which in turn trigger viral reactivation and impair NK cell antiviral activity, ultimately promoting viral spread (Wang et al., 2009; Liu et al., 2018; Wang et al., 2023). Notably, PAD4's substrate specificity for histones (H2A, H2B, H3, H4), p53, and STAT3 further supports its conserved role in chromatin remodeling and immune dysregulation across diverse viral infections (Pasquero et al., 2022).

### 3.3 Spatial compatibility and regulation of viral replication

**Figure 4:**
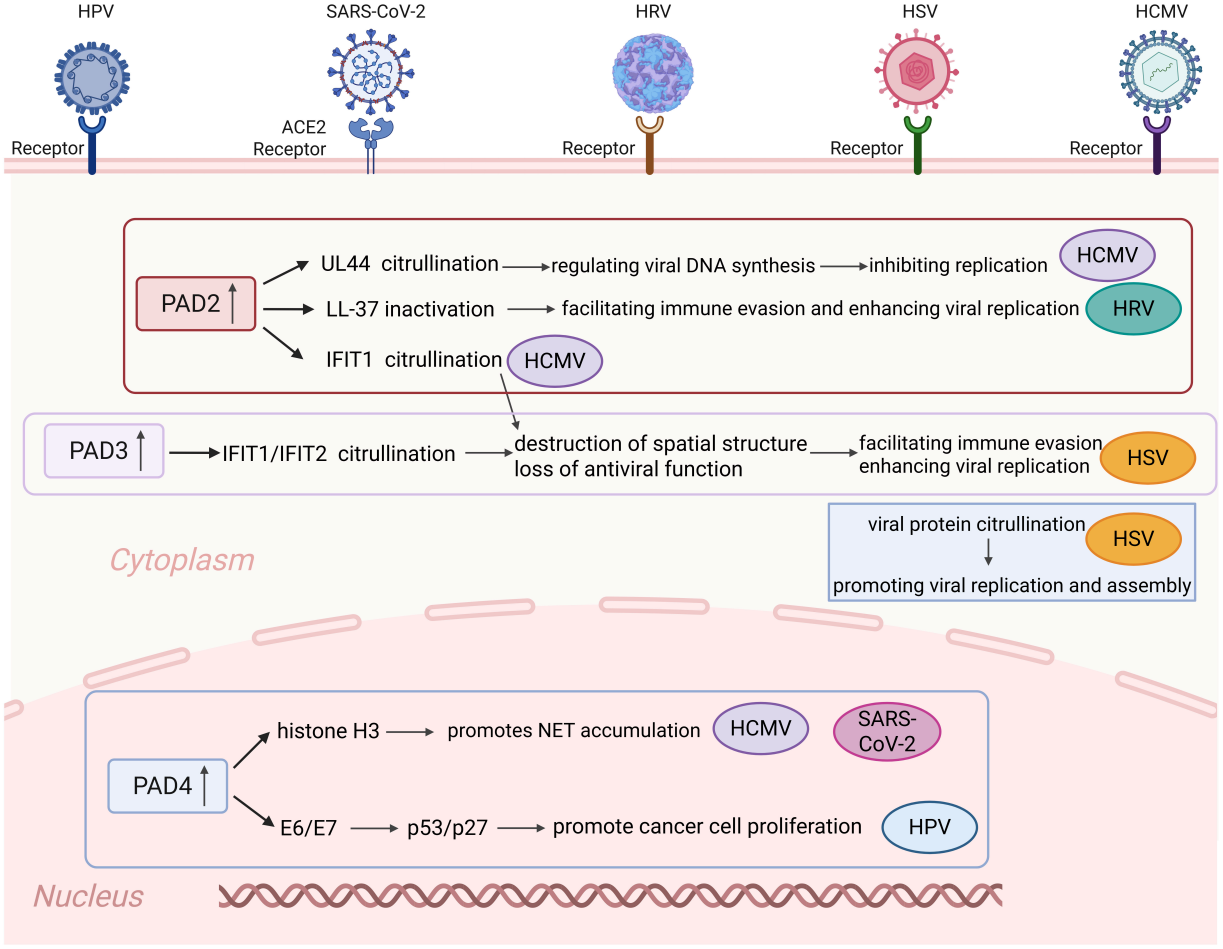
Multifaceted roles of PADs in viral infections. PAD isoforms (PAD2, PAD3, PAD4) mediate distinct effects on viral pathogenesis through protein citrullination in a virus- and compartment-specific manner. In the cytoplasm, upregulated PAD2 negatively regulates HCMV replication via UL44 citrullination, while citrullination of IFIT1 (HCMV) and LL-37 (HRV) impairs antiviral immunity and enhances replication. PAD3, upregulated during HSV infection, citrullinates IFIT1/IFIT2 to abrogate antiviral function, and directly modifies viral proteins to promote replication and assembly. In the nucleus, PAD4 citrullinates histone H3 to drive neutrophil extracellular trap (NET) formation in HCMV and SARS-CoV-2 infection, while citrullination of HPV oncoproteins E6/E7 dysregulates p53/p27 to promote cancer cell proliferation. Created with BioRender (BioRender.com).

Beyond immune escape and chromatin remodeling, the spatial compatibility between PAD isoforms and viral replication compartments, as well as the direct regulation of viral replication stages, constitutes another shared mechanism among diverse viruses. HRV replication-encompassing polyprotein synthesis, non-structural protein function, structural protein assembly, and viral particle release-occurs primarily in the cytoplasmic matrix and endoplasmic reticulum, a subcellular localization that overlaps with that of PAD2, providing a spatial basis for HRV's preferential utilization of PAD2 (Mello et al., 2014; Li et al., 2024). Similarly, HCMV's PAD2 and PAD4, which are upregulated during early infection, catalyze the citrullination of the viral polymerase accessory protein UL44 (Tian et al., 2020), thereby regulating viral DNA long-chain synthesis and inhibiting replication (Reyda et al., 2014); PAD2's localization in the cytoplasmic matrix and endoplasmic reticulum also overlaps with that of HCMV early (pp65) and late (gB) proteins, further supporting its role in HCMV replication (Reyda et al., 2014). HSV-1 and HSV-2 both upregulate PAD3, which promotes viral replication and assembly-evidenced by viral protein citrullination during both early and late infection stages, as well as the ability of broad-spectrum PAD inhibitors to suppress HSV-1 replication (Pasquero et al., 2024). For SARS-CoV-2, the spike protein binds to host ACE2 receptors to upregulate PAD4, initiating a cascade that induces NET secretion, reduces fibrinogen levels, and contributes to fibrinolysis and thrombosis-pathological processes closely linked to viral replication and pathogenesis (Zhang et al., 2020; Adriana Gutiérrez-Pérez et al., 2025). Collectively, these findings demonstrate that diverse viruses converge on PAD-mediated citrullination as a common strategy to manipulate host cellular processes for their own survival and replication (Figure 4).

### 3.4 Viral modulation of calcium homeostasis

Different viruses exhibit significant variations in genomic structure, infection routes, and host cell tropism. Consequently, their molecular mechanisms for regulating host calcium homeostasis, target sites, and patterns of Ca^2+^ concentration disruption also differ markedly (Qu et al., 2022). Following HPV infection, the oncogenic E6 and E7 proteins it encodes function as key molecules regulating calcium homeostasis. The E7 protein binds to host cell calmodulin (CaM), inhibiting CaM-dependent protein kinase II (CaMKII) activity and reducing Ca^2+^ release from the endoplasmic reticulum calcium pool. The E6 protein activates the PI3K-Akt signaling pathway, upregulates the expression of the plasma membrane calcium ATPase (PMCA), promotes intracellular calcium ion efflux, and further stabilizes intracellular calcium homeostasis (Chen et al., 1995). HSV (primarily HSV-1 and HSV-2) exhibits “stage-specific” and “receptor-dependent” regulation of calcium homeostasis (Cheshenko et al., 2014). This process centers on viral glycoproteins binding to host cell integrin receptors, activating Ca^2+^ signaling pathways to facilitate viral entry and intercellular spread. During the early stages of HSV infection (adsorption and entry), the viral envelope glycoprotein H (gH) binds to the host cell surface integrin αvβ3, activating focal adhesion kinase (FAK) phosphorylation. This subsequently activates the PLC-IP_3_ signaling pathway, promoting IP_3_R-mediated Ca^2+^ release from the ER calcium pool (Azab et al., 2015). This leads to a transient increase in intracellular Ca^2+^ concentration, which is essential for viral envelope fusion with the cell membrane and the entry of the viral capsid into the cytoplasm. The structural protein M of SARS-CoV-2 can synergize with the E protein to selectively influence Ca^2+^ release from the endoplasmic reticulum and the endoplasmic reticulum-mitochondria contact points, thereby affecting Ca^2+^ transients (Poggio et al., 2023). Abnormally elevated intracellular Ca^2+^ concentrations not only provide energy for viral replication, assembly, and release but also activate the NLRP3 inflammasome. This promotes pro-inflammatory cytokine secretion, triggering systemic inflammatory responses-a key mechanism underpinning severe pneumonia caused by SARS-CoV-2 infection.

### 3.5 PAD-mediated citrullination: a differential regulatory strategy between DNA and RNA viruses

DNA viral infections predominantly trigger the activation of PAD2, PAD3, and PAD4, with slight variations in subtype preference noted among different DNA viruses. As an example, HSV-1 infection markedly enhances the expression of PAD2, PAD3, and PAD4 via transcriptional regulatory pathways (Pasquero et al., 2023); Upon HCMV infection in primary human fibroblasts, members of the PAD family are predominantly activated, which triggers the citrullination of host proteins and facilitates viral adaptation. In cervical epithelial cells infected with HPV, PAD4 expression is notably upregulated in a manner that correlates with disease progression (Chang et al., 2009). RNA viruses display a more restricted pattern of PAD subtype activation. Existing studies indicate that they predominantly target PAD4, with minimal or no significant regulation of other PAD isoforms. This subtype-specific activation pattern is well exemplified by SARS-CoV-2 (Bouaziz et al., 2020).

Following infection with DNA and RNA viruses, the repertoires of host and viral proteins targeted for citrullination differ substantially. These distinct substrate classes reflect the divergent mechanisms by which these two viral groups utilize PAD-mediated citrullination to support replication and evade host immunity. Upon DNA virus infection, citrullinated substrates consist of host immune regulators, viral structural proteins, and host metabolic proteins. Notably, the interferon-induced proteins IFIT1 and IFIT2 are key host substrates. As an example, HSV-1 infection drives extensive citrullination of IFIT1 and IFIT2. Genetic knockout of these proteins significantly boosts HSV-1 replication, implying that DNA viruses inhibit their antiviral activity through citrullination to achieve immune evasion (Pasquero et al., 2023). Following HCMV infection, IFIT1 still acts as the main citrullination substrate. *In vitro* studies have verified that IFIT1 citrullination impairs its capacity to bind 5′-ppp RNA, thereby reducing its antiviral activity and creating a favorable environment for viral replication (Griffante et al., 2021). Limited clinical evidence suggests that during HPV infection, PAD4-mediated citrullination regulates the functions of host tumor suppressors including p53 and p21. Meanwhile, this process also affects the expression of the HPV oncoproteins E6 and E7, ultimately driving malignant transformation of cervical epithelial cells (Albano et al., 2024).

Moreover, DNA viruses can induce histone citrullination in host cells to modulate transcriptional programs, thereby establishing a permissive intracellular environment for viral replication. However, upon RNA virus infection, citrullination primarily targets host immune-associated proteins, particularly components of the interferon signaling pathway, with minimal modification of viral proteins. Following infection with RNA viruses including SARS-CoV-2 and HRV, IFIT1 serves as the primary substrate for host protein citrullination. By compromising the antiviral activity of IFIT1, these RNA viruses successfully evade host immune surveillance. Collectively, these distinct regulatory strategies offer new potential targets and mechanistic insights for the differential diagnosis of DNA and RNA virus infections, as well as for the development of antiviral therapeutics. Given the important role of PAD in viral infection, inhibitors and activators targeting this enzyme have emerged as potential therapeutic strategies against viral diseases (Mello et al., 2014; Elliott et al., 2021; Griffante et al., 2021; Albano et al., 2024; Pasquero et al., 2024).

## 4 PAD inhibitors and activators: development and biological functions in viral infections

### 4.1 PAD inhibitors

**Figure 5:**
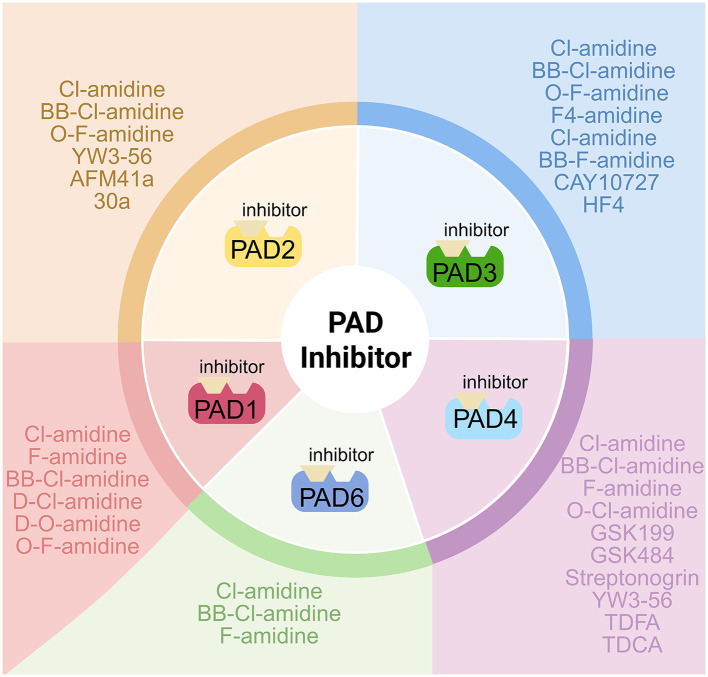
Comparative analysis of PAD isoenzyme inhibitors. The figure presents a comparative analysis of the inhibitory profiles of multiple PAD inhibitors against individual PAD isoforms. Created with BioRender (BioRender.com).

The close association between the citrullination process and various human diseases, along with PAD gene overexpression in malignancies, has prompted the development of a series of PAD inhibitors (Figure 5). The initial generation of PAD inhibitors were irreversible pan-PAD enzyme inhibitors that were designed based on the PAD active site. Subsequent development has been driven by the substrate specificity of PAD enzymes, resulting in the creation of specific PAD inhibitors that target different PAD types (Barasa and Thompson, 2023; Mansouri et al., 2024). These inhibitors represent potential intervention tools for associated pathologies (Witalison et al., 2015; Yuzhalin, 2019; Zhu et al., 2021), with several demonstrating notable antiviral efficacy in preclinical studies (Arisan et al., 2020; Griffante et al., 2021; Pasquero et al., 2022; Albano et al., 2024; Pasquero et al., 2024).

A number of reversible PAD inhibitors, such as chloramphenicol, minocycline, ruthenium red, sanguinarine, GSK199, and GSK484, were identified, though their exact mechanisms remain elusive. Among these, GSK199 and GSK484 are highly potent and selective PAD4 inhibitors (Mondal and Thompson, 2019), with GSK199 shown to significantly suppress the replication of human coronavirus such as HCoV-OC43 and SARS-CoV-2 (Pasquero et al., 2022). Different small-molecule inhibitors displayed specificities: D-Cl-amidine and D-O-F-amidine are directed toward PAD1; F4-amidine, Cl-4-amidine, and BB-F-amidine target PAD3; while BB-Cl-amidine, F-amidine, and Cl-amidine functioned as pan-PAD inhibitors, affecting multiple subunits, Cl-amidine and BB-Cl-amidine effectively inhibit the replication of HCMV, HSV-1 and HSV-2 (Griffante et al., 2021; Pasquero et al., 2023, 2024). O-Cl-amidine selectively targeted PAD1/PAD4 (Causey et al., 2011), whereas TDFA was a PAD4-specific inhibitor (Luo et al., 2006; Knight et al., 2015; Mondal and Thompson, 2019). Irreversible inhibitors, such as streptomycin (a potent PAD4 inhibitor) and NSC95397, were also identified (Mondal and Thompson, 2019). YW3-56 is a PAD2 and PAD4-selective inhibitor that hinders the proliferation of cancer cells at low concentrations (Wang et al., 2012). Clinical studies confirmed the efficacy of certain inhibitors. For instance, TDFA demonstrates notable therapeutic efficacy in treating acute lung injury in mice post-SARS-CoV-2 infection (Elliott et al., 2021). The PAD3 inhibitor CAY10727 inhibits HSV-2 infection in HFFS cells *in vitro* by reducing cytopathic effects and viral replication (Pasquero et al., 2024). Many inhibitors exhibited isoenzyme selectivity for precise targeting, CAY10727 and HF4 selectively inhibit PAD3 without affecting other subtypes and potently reduce HSV-1 replication, with CAY10727 also inhibiting HSV-2 (Table 1) (Pasquero et al., 2023). Continued preclinical and clinical investigation validates the promise of these agents as targeted therapeutics for both inflammatory and infectious conditions.

**Table 1:** PAD inhibitors and mechanisms of action.

PAD inhibitors	Characteristics	Mechanism/functional effect	Reference(s)
GSK199, GSK484	Reversible, potent and selective PAD4 inhibitors	Suppress replication of human coronaviruses (HCoV-OC43, SARS-CoV-2); decreases pulmonary NET accumulation and improves clinical symptoms in a mouse model of SARS-CoV-2 infection	Mondal and Thompson, 2019; Pasquero et al., 2022; Bonilha et al., 2025
Streptonigrin	Irreversible PAD inhibitor, selective PAD4 inhibitor	Contains an α, β-unsaturated carbonyl functionality and modifies the active site cysteine	Mondal and Thompson, 2019
Cl-amidine	Irreversible pan-PAD inhibitor	Effectively inhibits NETosis and replication of HCMV, HSV-1, HSV-2; Reduces disease activity in collagen-induced arthritis, ameliorates DSS-induced colitis, suppresses AOM/DSS-driven tumor progression; improves vascular and renal function while alleviating cutaneous lesions in MRL/lpr mice	Luo et al., 2006; Chumanevich et al., 2011; Wang et al., 2012; Knight et al., 2015; Witalison et al., 2015; Mondal and Thompson, 2019; Griffante et al., 2021; Pasquero et al., 2023, 2024
F-amidine	Irreversible pan-PAD inhibitor	Inhibits PAD4-dependent citrullination of p300	Mondal and Thompson, 2019
F4-amidine, Cl4-amidine	Irreversible pan-PAD inhibitor, PAD3-selective	Selective inhibition of PAD3	Mondal and Thompson, 2019
O-F-amidine	Irreversible PAD inhibitor, PAD1-selective	Blocks the citrullination of histone H3 in HL-60 cells	Mondal and Thompson, 2019
O-Cl-amidine	Irreversible PAD inhibitor, PAD1/PAD4-selective	Blocks the citrullination of histone H3 in HL-60 cells	Causey et al., 2011; Mondal and Thompson, 2019
BB-F-amidine	Irreversible PAD inhibitor, PAD3-selective	Exhibits similar *pp* potency as Cl-amidine and F-amidine	Knight et al., 2015; Mondal and Thompson, 2019
BB-Cl-amidine	Irreversible pan-PAD inhibitor	Effectively inhibits NETosis	Knight et al., 2015; Mondal and Thompson, 2019
AFM41a	Novel PAD inhibitor, PAD2-selective	Protects against *Pseudomonas aeruginosa* (PA) pneumonia-induced sepsis	Dong et al., 2024
YW3-56	Novel PAD inhibitor, PAD2 and PAD4-selective	Hinders proliferation of cancer cells at low concentrations	Wang et al., 2012
TDFA	PAD4-specific inhibitor	Demonstrates therapeutic efficacy in treating acute lung injury post-SARS-CoV-2 infection	Luo et al., 2006; Knight et al., 2015; Mondal and Thompson, 2019; Elliott et al., 2021
CAY10727	PAD3-selective inhibitor	Inhibits HSV-2 infection (by reducing cytopathic effects and viral replication) and potently reduces HSV-1 replication; reduces cytopathic effects and viral replication in HFF cells	Pasquero et al., 2023, 2024

PAD inhibitors have shown significant biological activity across multiple preclinical disease models. Cl-amidine reduces disease activity in collagen-induced arthritis, ameliorates dextran sulfate sodium (DSS)-induced colitis, and suppresses azoxymethane (AOM)/DSS-driven tumor progression; in MRL/lpr mice, Cl-amidine and BB-Cl-amidine improve vascular and renal function while alleviating cutaneous lesions (Chumanevich et al., 2011; Knight et al., 2015; Witalison et al., 2015). The PAD2-selective inhibitor AFM41a markedly enhances survival in sepsis models (Dong et al., 2024). In SARS-CoV-2 infection models, GSK484 decreases pulmonary NET accumulation and improves clinical symptoms, but may carry a potential risk of impaired T cell responses (Bonilha et al., 2025).

Safety evaluation revealed no significant drug-related adverse effects in mice following 14 days of daily Cl-amidine administration, and no obvious systemic immunosuppression was detected in the 35-day chronic treatment study (Chumanevich et al., 2011; Willis et al., 2011). Consistently, administration of YW3-56 in a mouse sarcoma S-180 xenograft model did not result in significant adverse effects on the vital organs of the mice and exhibited an extremely low incidence of overall toxic and side effects (Wang et al., 2012). Compared with Cl-amidine, BB-Cl-amidine exhibits higher cellular potency (half-maximal effective concentration EC_50_: 8.8 μM vs. >200 μM) and a longer *in vivo* half-life (1.75 h vs. 15 min) (Knight et al., 2015). Notably, while GSK484-mediated PAD4 inhibition mitigates NET-driven lung injury in a mouse model of SARS-CoV-2 infection, it may adversely impact adaptive immune responses by impairing dendritic cell antigen presentation and T cell interleukin-2 (IL-2) signaling (Bonilha et al., 2025).

Currently, the majority of reported inhibitors are either pan-PAD inhibitors or only active against a single isoform (e.g., PAD4 or PAD2), with limited subtype-specific regulation. Although several naphthalene- or quinoline-based scaffolds can improve selectivity toward PAD1/PAD4, they still fail to fully eliminate off-target inhibition toward other isoforms (Dakin et al., 2025). Off-target effects typically arise from non-specific interactions between therapeutic agents and non-target biomolecules. Findings from mouse models have demonstrated that such off-target liabilities may be partially alleviated via structure-based optimization strategies (Salzmann et al., 2024). For example, the potent and selective PAD4 inhibitor GSK484 exhibits no agonist activity toward HDACs 1-11 at concentrations up to 100 μg/ml, confirming that structural optimization can effectively reduce off-target risks (Koushik et al., 2017). Despite these advances, completely eliminating off-target risks remains challenging, necessitating continued progress in designing subtype-specific drug interactions.

### 4.2 PAD activators

Although diverse citrullination inhibitors exist, PAD activators are less studied. Zhang X. et al. confirmed that Demethoxycurcumin (DMC) acts as a specific PAD2 activator (Zhang et al., 2023). DMC binds to the E352 residue of PAD2, promoting its interaction with histone H3 and activating the citrullination reaction. This compound provides a crucial research tool for exploring the molecular mechanisms and functional roles of PAD2 and citrullination modification in disease progression.

DMC is currently the only reported novel direct and selective activator of PAD2, providing an important tool for investigating the functional role of this enzyme during viral infection. Combining DMC treatment with PAD2 knockdown allows effective validation of its regulatory function in viral infection. To date, the development of activators has lagged considerably behind that of inhibitors, largely because research has focused on autoimmune diseases closely associated with aberrant citrullination, where inhibitors exhibit more defined therapeutic potential. Furthermore, the screening of activators presents substantial technical challenges, and enhancing PAD enzymatic activity *in vivo* carries inherent safety concerns (Zhang et al., 2023).

## 5 Conclusions and future perspectives

This review not only reaffirms the established link between PADs, citrullination, and viral infection but also explains why specific viruses preferentially utilize particular PADs and how calcium homeostasis is regulated in different viral types, thereby enhancing our understanding of the underlying mechanisms. As emphasized by Qu et al. (2022), different viruses employ distinct strategies to disrupt host calcium homeostasis. For instance, HPV relies on its oncogenic proteins E6 and E7; E7 binds calmodulin to inhibit CaMKII activity and reduce ER calcium release, while E6 activates the PI3K-Akt pathway to upregulate PMCA and promote calcium efflux (Chen et al., 1995). In contrast, HSV exhibits “stage-specific” regulation, where glycoprotein gH binds integrin αvβ3 to activate the PLC-IP3 pathway, triggering IP3R-mediated Ca^2+^ release from the ER to facilitate viral entry (Cheshenko et al., 2014; Azab et al., 2015). SARS-CoV-2, meanwhile, utilizes its structural M and E proteins to modulate Ca^2+^ release at ER-mitochondria contact points, with elevated Ca^2+^ not only supporting viral replication but also activating the NLRP3 inflammasome, thereby driving severe inflammatory responses (Poggio et al., 2023). Beyond these virus-encoded mechanisms, disruptions in calcium homeostasis can also arise from host cell death pathways and pathogen-mediated membrane damage. As reported by Alghamdi et al., programmed cell death in keratinocytes leads to inactivation of the PMCA calcium pump, resulting in intracellular calcium accumulation and subsequent PAD activation. Similarly, in persistently activated macrophages, the loss of plasma membrane integrity during apoptosis or necrosis facilitates calcium influx. This phenomenon is also exploited by certain pathogens, which disrupt cell membrane structure via specific virulence factors such as the LtxA toxin, thereby inducing calcium influx and aberrant PAD activation (Alghamdi et al., 2019). Furthermore, it addresses the scientific challenge of why activators lag behind inhibitors in development. However, extant research is subject to certain limitations: Firstly, the field of PAD-virus interactions remains relatively limited, lacking a systematic and comprehensive research framework. Secondly, although current studies suggest that PAD dysregulation plays a critical role in viral infection, this conclusion remains a preliminary hypothesis, lacking sufficient *in vitro* and *in vivo* experimental data support. Furthermore, the specific molecular mechanisms underlying these effects such as how viruses regulate PAD expression and how PAD-mediated citrullination precisely affects the viral replication cycle) have not been elaborated in depth.

### 5.1 Evaluation of research findings

*In vitro* studies, such as those involving SARS-CoV-2 and PAD4 (PAD2 and HRV) and direct molecular interactions (HSV-1 ICP27 and PAD3), offer the advantage of defining virus-specific PAD isoform activation. Animal studies are crucial for validating the biological significance of *in vitro* findings *in vivo*, particularly for coronaviruses and herpesviruses (Pasquero et al., 2022, 2024). For instance, mouse SARS-CoV-2 infection models confirmed that PAD4 inhibition reduces NET accumulation and lung injury (Bonilha et al., 2025). Clinical data on viral-PAD interactions remain the most limited yet translationally critical evidence, offering advantages such as direct relevance to human viral infections and the potential to identify clinical biomarkers (e.g., CitH3 as a biomarker for severe COVID-19).

However, most mechanistic conclusions lack validation in physiologically relevant animal models, and clinical data are limited for only a few viral families. Secondly, the PAD isoforms and substrates in mouse animal models exhibit distinct properties compared to their human orthologs, and most viral animal models fail to reproduce the affinity and disease severity of human viruses. Third, existing clinical data are almost entirely correlational, lacking interventional studies to confirm the causal role of PADs in human viral diseases.

### 5.2 Future directions

Based on the current research status, several key unresolved issues in this field urgently require further exploration: (1) It is necessary to systematically analyze the expression regulation patterns and selective activation mechanisms of various PAD isoforms during infection with different viruses, and identify the direct interaction targets between viral proteins and PAD isoforms; (2) In-depth investigation should be conducted to clarify the specific regulatory networks through which PAD-mediated citrullination modulates viral protein function, viral genome replication, and host antiviral immune responses, revealing the role of imbalanced interactions among “virus-PAD-host” in the occurrence and development of diseases; (3) The antiviral efficacy and safety of PAD inhibitors need to be verified in different viral infection models to evaluate their potential as broad-spectrum antiviral therapeutic agents; (4) Clinical sample research should be expanded to determine the correlation between PAD expression levels, citrullinated protein profiles, and disease severity, treatment response, and prognosis in patients with viral infections, providing a basis for the development of clinical diagnostic markers and therapeutic targets. These research directions will provide important support for improving the theoretical system of PAD-virus interactions and promoting the development of novel antiviral therapeutic.
